# COVID-19 in humanitarian settings: documenting and sharing context-specific programmatic experiences

**DOI:** 10.1186/s13031-020-00321-w

**Published:** 2020-11-19

**Authors:** Neha S. Singh, Orit Abrahim, Chiara Altare, Karl Blanchet, Caroline Favas, Alex Odlum, Paul B. Spiegel

**Affiliations:** 1 Health in Humanitarian Crises Centre, London School of Hygiene and Tropical Medicine, London, UK; 2 Center for Humanitarian Health, Johns Hopkins Bloomberg School of Public Health, Baltimore, USA; 3 Geneva Centre of Humanitarian Studies, University of Geneva and the Graduate Institute, Geneva, Switzerland

**Keywords:** COVID-19, Humanitarian, Refugee, Conflict, Crisis, Platform

## Abstract

Humanitarian organizations have developed innovative and context specific interventions in response to the COVID-19 pandemic as guidance has been normative in nature and most are not humanitarian specific. In April 2020, three universities developed a COVID-19 humanitarian-specific website (www.covid19humanitarian.com) to allow humanitarians from the field to upload their experiences or be interviewed by academics to share their creative responses adapted to their specific country challenges in a standardised manner. These field experiences are reviewed by the three universities together with various guidance documents and uploaded to the website using an operational framework. The website currently hosts 135 guidance documents developed by 65 different organizations, and 65 field experiences shared by 29 organizations from 27 countries covering 38 thematic areas. Examples of challenges and innovative solutions from humanitarian settings are provided for triage and sexual and gender-based violence. Offering open access resources on a neutral platform by academics can provide a space for constructive dialogue among humanitarians at the country, regional and global levels, allowing humanitarian actors at the country level to have a strong and central voice. We believe that this neutral and openly accessible platform can serve as an example for future large-scale emergencies and epidemics.

## Background

As the COVID-19 pandemic continues to spread across the world, its negative effects among persons affected by humanitarian emergencies are becoming increasingly apparent. High population density, limited access to preventative and curative health services, poor water, sanitation and hygiene services, poor governance, distrust of authorities, and increasing stigma and discrimination are among the many risk factors that make the prevention and management of COVID-19 particularly challenging in such settings [1]. These include conflict-affected countries such as South Sudan, Yemen and Syria, and forced displacement settings such as refugees in Bangladesh and Lebanon and internally displaced persons in Ethiopia and the Democratic Republic of Congo (DRC). The direct and indirect impacts of COVID-19 and the response to control its spread in these environments are currently under-documented and under-researched [2–4].

COVID-19 guidance primarily focuses on high income countries, that have become the epicentre of the pandemic, and thus far has often not necessarily been as relevant or applicable to humanitarian settings where living and working conditions as well as socio-cultural environments are very different, and where local health systems are already weakened by existing humanitarian crises. Furthermore, such guidance developed for humanitarian settings [5, 6] cannot by their nature be context-specific, and thus humanitarian organizations at the country level have initiated their own innovative interventions to respond to the specific challenges they have been experiencing [7].

## Process

At the beginning of the pandemic, the Center for Humanitarian Health (Johns Hopkins University), the Health in Humanitarian Crises Centre (London School of Hygiene and Tropical Medicine), and the Geneva Centre of Humanitarian Studies (University of Geneva, The Graduate Institute) received a multitude of requests from governments, United Nations agencies and non-governmental organizations (NGOs) working in humanitarian settings – all of them with a similar thread – *how can we operationalise evolving global COVID-19 guidance to our specific context*?

**Fig. 1:**
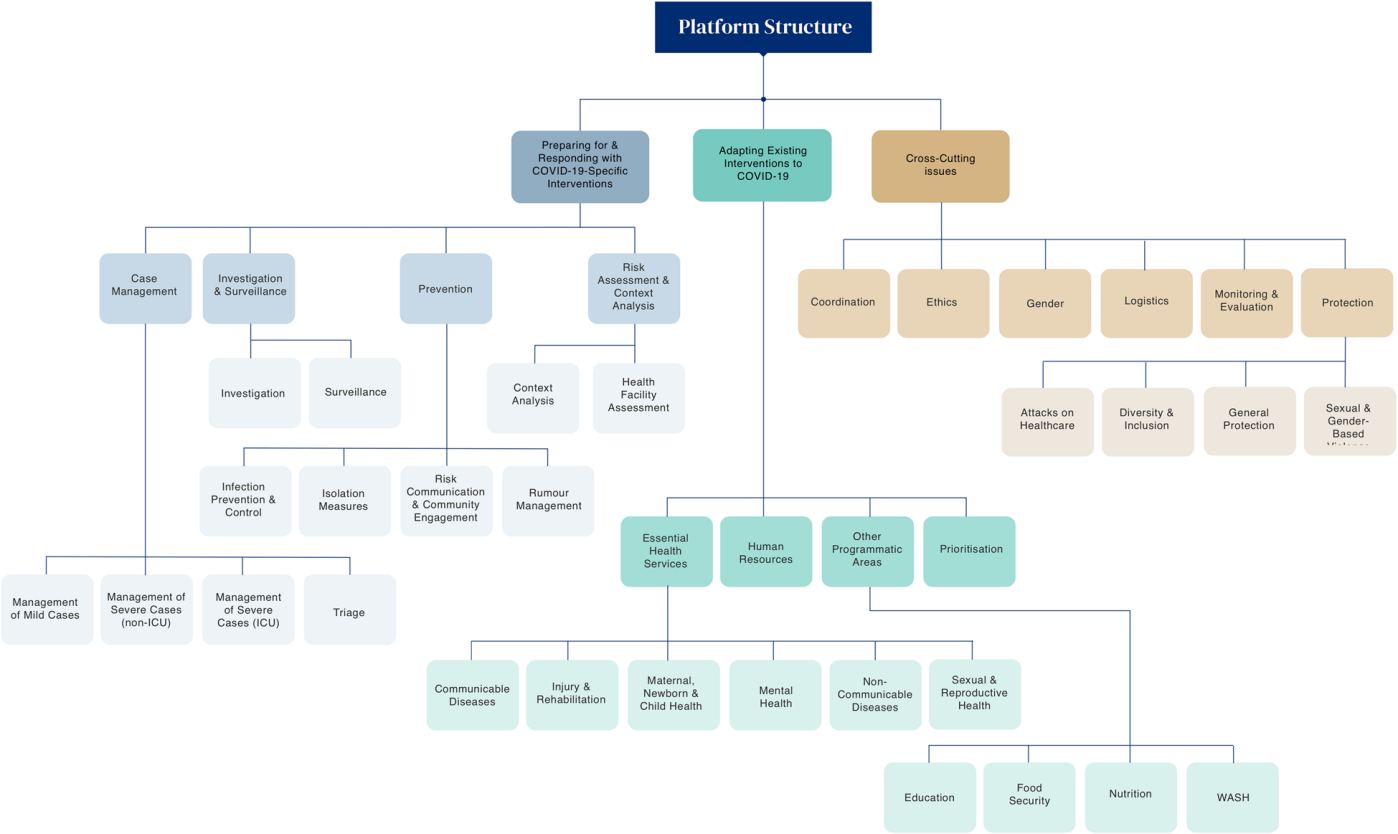
COVID-19 Humanitarian Platform Framework

The three universities decided to facilitate sharing and learning across humanitarian settings, by creating an open access online platform to compile both existing relevant guidance and field experiences from frontline responders to understand: (i) how to prepare and respond to the pandemic; ii) how to adapt existing interventions to ensure continuity of services in a COVID-19 context; and iii) how to address cross-cutting issues in a COVID-19 context. We sought to present COVID-19 guidance and field experiences within an operational framework that would allow users to locate information by specific humanitarian activity areas within these three categories (see Fig. 1).

Within two weeks of this decision, the www.covid19humanitarian.com platform was up and running on April 20, 2020. This unique platform allows humanitarian actors to learn from one another about individual programme responses, adaptations and innovations collected and reviewed in a standardised manner. It also provides easy access to COVID-19 guidance produced at global and regional levels according to an operational framework (Fig. 1).

The two domains, guidance and field experiences were chosen to present high-level recommendations alongside the realities of program implementation at the field-level. The collection and review of both domains underwent two processes. For the selection of guidance, a team of reviewers from the three universities assessed each document for applicability to humanitarian or low-income/fragile settings, reputability, potential bias, evidence base (including experiential), and practicality. Guidance documents were reviewed and uploaded weekly. For field experiences, an interview template was used to gather experiences from field actors in a standardized manner, and an upload template was used to share experiences so that information may be compared across activities and between contexts. Users are also able to submit their own experiences by completing the same upload template available on the platform. Uploads through the platform are reviewed by the research team for appropriateness. The platform allows users to search through experiences by country, setting (urban, rural, mixed), target population (e.g. refugees, internally displaced populations, etc.), and category/section/area of the operational framework. Users are also able to search the site globally with keywords.

## Results

The www.covid19humanitarian.com website currently hosts 135 guidance documents developed by 65 different organizations that have been published after undergoing review. Of the 135 documents, 46 (34%) provide guidance on how to prepare and respond to COVID-19, 52 (39%) on how to adapt existing interventions to ensure continuity of implementation, and 37 (27%) refer to cross-cutting issues. United Nations agencies have produced nearly 70% of the guidance on the website (92 documents) with the World Health Organization and UNICEF accounting for 36 and 18 guidance documents, respectively.

Since April 2020, the team has conducted 77 qualitative interviews with humanitarian workers from local civil society and grassroots initiatives, international and national NGOs and UN agencies based primarily in the field, with plans to continue to conduct interviews over the course of the pandemic. To date, this process has generated 56 field experience summaries that underwent a standardized review by the three universities, and then were uploaded to the platform. Most organizations have implemented a combination of multiple interventions and adaptations to respond to COVID-19, but to collect an informative level of detail, interviews focused on specific framework areas (Fig. 1), most frequently triage (*n* = 7 interviews), risk communication (*n* = 7), context analysis (*n* = 6), food security, particularly food, cash and voucher distributions (*n* = 5), nutrition (*n* = 5), mental health and psychosocial support (*n* = 5), infection prevention and control (*n* = 4),, education (*n* = 3), and sexual and gender-based violence (SGBV) (*n* = 3). As of August 2020, Lebanon (*n* = 7 interviews), Pakistan (*n* = 6), South Sudan (*n* = 5), Afghanistan (*n* = 4),, Bangladesh (*n* = 3), Colombia (*n* = 3), DRC (*n* = 3), Greece (*n* = 3), Haiti (*n* = 3), Jordan (*n* = 3), Libya (*n* = 3), Nigeria (*n* = 3) and Yemen (*n* = 3) are the most frequently covered countries (see Table 1).

**Table 1:** Field Experiences per Country according to Framework Area

Interviews conducted per country																																				
Framework area	Lebanon	Pakistan	South Sudan	Afghanistan	Libya	Greece	Colombia	Nigeria	Haiti	Yemen	Bangladesh	D.R. Congo	Jordan	Syria	France	Kenya	Mexico	Cen. Af. Rep.	Palestine	Myanmar	Iraq	USA	Uganda	Malaysia	Indonesia	Philippines	Turkey	Somalia	Ukraine	Ethiopia	Nepal	Sudan	Italy	India	Tanzania	Total
**Adapting Existing Interventions to COVID-19**	**4**	**3**	**1**	**3**		**1**	**1**	**1**			**1**	**2**	**2**		**1**	**1**		**1**	**1**	**1**			**1**			**1**				**1**	**1**	**1**			**1**	**30**
**Essential health services**	**3**	**2**	**1**	**1**		**1**						**1**	**1**			**1**							**1**			**1**									**1**	**14**
Mental health	2											1														1									1	5
Unspecified		1				1																														2
Sexual reproductive health		1																					1													2
Maternal and child health			1	1																																2
Non-communicable diseases	1												1																							2
Communicable diseases																1																				1
**Other programmatic areas**	**1**	**1**		**2**			**1**	**1**			**1**	**1**	**1**					**1**	**1**	**1**										**1**	**1**	**1**				**15**
Food security								1				1							1											1		1				5
Nutrition		1		2							1							1																		5
Education	1												1																		1					3
Wash							1																													1
Unspecified																				1																1
**Prioritization**															**1**																					**1**
**Cross-cutting issues**	**3**	**1**					**1**		**1**								**2**					**1**			**1**											**10**
**Coordination**		**1**																							**1**											**2**
**Ethics**	**1**																																			**1**
**Logistics**																						**1**														**1**
**Protection**	**2**						**1**		**1**								**2**																			**6**
Sexual and gender based violence	2								1																											3
General protection																	1																			1
Attacks on healthcare							1																													1
Unspecified																	1																			1
**Preparing for and responding to COVID-19**		**2**	**4**	**1**	**3**	**2**		**2**	**2**	**2**	**2**	**1**		**1**	**1**	**1**		**1**	**1**	**1**	**1**						**1**	**1**	**1**				**1**			**32**
**Case management**		**1**	**2**			**1**					**1**			**1**						**1**	**1**							**1**								**9**
Triage			2			1					1									1	1							1								7
Unspecified														1																						1
Management of mild cases		1																																		1
**Investigation and surveillance**			**1**	**1**												**1**																				**3**
Surveillance				1												1																				2
Investigation			1																																	1
**Prevention**		**1**			**1**	**1**		**2**	**2**	**2**	**1**							**1**	**1**														**1**			**13**
Risk communication and community engagement		1			1			1	2	2																										7
Infection prevention and control						1					1							1															1			4
Rumor management								1																												1
Isolation measures																			1																	1
**Risk assessment and context analysis**			**1**		**2**							**1**			**1**												**1**		**1**							**7**
Context analysis			1		2							1			1												1									6
Health facility assessment																													1							1
**Unspecified**							**1**			**1**				**1**										**1**										**1**		**5**
**Total**	**7**	**6**	**5**	**4**	**3**	**3**	**3**	**3**	**3**	**3**	**3**	**3**	**2**	**2**	**2**	**2**	**2**	**2**	**2**	**2**	**1**	**1**	**1**	**1**	**1**	**1**	**1**	**1**	**1**	**1**	**1**	**1**	**1**	**1**	**1**	**77**

We now present an illustration of what we have documented from two areas in the platform’s framework, triage and SGBV, to show how useful it can be for programs to collect and compare field experiences from various contexts.

### Triage

Triage is an essential step in the clinical pathway that generally aims to prioritize treatment of patients according to urgency. The variety of outcomes caused by COVID-19 infection, ranging from low acuity to severely ill cases highlights the importance of prioritizing care to those who need it the most. In the context of COVID-19, triage also aims to minimize nosocomial transmission.

Given its importance for an effective health system response, COVID-19 triage systems were established rapidly across the world and adapted to local health facility conditions. Experiences reported by humanitarian actors in Myanmar, South Sudan, DRC and Somalia share many similarities such as the use of temperature screening at the health facility entrance as first alert sign, or the existence of national protocols.

Beyond the standard procedures described in global guidance, several context specific differences have been identified such as case definitions, both in terms of which signs are used to define a suspect case, and at which step symptoms and epidemiological risks are investigated. In settings where malaria is endemic and the prevalence of other infections is high, e.g. DRC or South Sudan, relying mainly on fever or cough to identify and isolate suspect cases can generate unnecessary burden and delays for investigation teams, while increasing the risk of cross contamination.

Community-based triage was reported in multiple contexts thus far, including in camps on the Greek islands, and rural border communities in South Sudan and in Somalia. These experiences involve community health workers engaging with community members to postpone non-urgent clinic visits. Yet, finding the balance between reducing risk of infection and ensuring continuity of essential services remains a challenge in many country experiences. As observed in other epidemics, excess morbidity and mortality for other non-COVID-19 causes are likely to exceed COVID-19 related deaths [8]. Another difference relates to isolating suspected and/or confirmed cases, specifically whether it is voluntary as in Somalia, DRC and South Sudan or compulsory as in Myanmar. This issue can be very sensitive in conflict-affected settings where trust in national authorities may be limited, thus, increasing the risk of people refusing to seek treatment and testing when sick. Finally, the integration of rapid tests to rule out other conditions, such as malaria, was only reported in South Sudan, and could serve as a good model for other countries.

### Sexual and gender-based violence

There has been alarming information on increased SGBV occurring against the backdrop of the COVID-19 outbreak globally. Many of the measures deemed necessary to control the spread of the virus (e.g. restricted movement and social isolation measures) are not only increasing SGBV-related risks and violence against women and girls, but also limiting survivors’ ability to distance themselves from their abusers as well as reducing and accessing external support [9].

Humanitarian actors’ experiences from Haiti and Lebanon on adapting SGBV programmes to COVID-19 share a number of similarities, including: (i) strong community mobilisation and support; (ii) adequate and sustained funding; (iii) creative use of media and digital technology including social media (e.g. WhatsApp) to reach target populations and support frontline workers; (iv) strong and sustained partnerships and coordination to enable dissemination of messages; and (v) mainstreaming SGBV into other programmes, e.g. printing a hotline number on the back of a World Food Program card for cash distributions in Haiti, where the vast majority of beneficiaries are female.

SGBV programming across contexts also differ in who delivers the intervention (e.g. fully women-led in Haiti; using social media influencers in Lebanon). Finally, the modality of implementation is also calibrated to the contexts, including their urban and rural characteristics. In Haiti, SGBV programmes in rural areas with limited internet and phone network coverage were delivered mostly in person while following physical distancing rules (e.g. via friendly spaces, community mobilisers), whereas in Lebanon, a more urbanised context, activities were mostly undertaken remotely (e.g. via e-awareness platforms, remote psychosocial and emotional support sessions), except for high risk populations.

## Conclusions

Supporting frontline humanitarian workers with context specific technical guidance is crucial in such challenging times as a pandemic, specifically when most guidance is primarily normative from the global level, and not created for a humanitarian context or quite broad to be include all types of humanitarian settings. Offering open access resources on a neutral platform where academics interview humanitarians from the field and write up their experiences in a standardised format, which are then reviewed by academics before being uploaded is an added value for the humanitarian community. Our platform aims to amplify country-level humanitarian actors’ voices by sharing their experiences widely and provides for a less top-down approach to the development of guidance. Through the collection of field experiences, this project has demonstrated the quick reactivity and creativity of humanitarian actors on the ground who are faced with vulnerable populations living in environments where the implementation of the COVID-19 recommended prevention and transmission control measures is challenging. These unique and standardised field experiences can serve as a basis for improved regional and global COVID-19 humanitarian guidance iterations. However, at present such a feedback loop does not appear to be occurring, since such humanitarian-specific guidance by the global agencies has not sufficiently evolved from the early days of the pandemic. Our goal is for this platform to provide a space for constructive dialogue among humanitarians at the country, region and global levels, allowing the humanitarian at the country level to have a strong and central voice and our group to engage with the Global Health Cluster to make sure these field-based experiences are used for future guidelines. We believe that this neutral and openly accessible platform can serve as an example for future large-scale emergencies and epidemics.

## Data Availability

Not applicable.

## References

[1] Truelove S, Abrahim O, Altare C (2020). The potential impact of COVID-19 in refugee camps in Bangladesh and beyond: a modeling study. PLoS Med.

[2] Alemi Q, Stempel C, Siddiq H, Kim E (2020). Refugees and COVID-19: achieving a comprehensive public health response. Bull World Health Organ.

[3] Alawa J, Alawa N, Coutts A, Sullivan R, Khoshnood K, Fouad FM (2020). Addressing COVID-19 in humanitarian settings: a call to action. Confl Health.

[4] Blanchet K, Alwan A, Antoine C, et al. Protecting essential health services in low-income and middle-income countries and humanitarian settings while responding to the COVID-19 pandemic. BMJ Glob Health. 2020;5(10):e003675. 10.1136/bmjgh-2020-003675 PMC7542611 33028701

[5] Interagency Standing Committee. Interim Guidance on Scaling-up COVID-19 Outbreak in Readiness and Response Operations in Camps and Camp-like Settings. https://interagencystandingcommittee.org/other/interim-guidance-scaling-covid-19-outbreak-readiness-and-response-operations-camps-and-camp. Accessed 6 Nov 2020.

[6] Ramalingam B, Singh N, Mahieu A, Blanchet K. Responding to COVID-19. Guidance for humanitarian agencies. ALNAP Rapid Learning Review London: ODI/ALNAP Available at: https://www.alnap.org/help-library/responding-to-covid-19-guidance-for-humanitarian-agencies. Accessed 6 Nov 2020.

[7] Editorial (2020). Humanitarian crises in a global pandemic. Lancet.

[8] Brolin Ribacke KJ, Saulnier DD, Eriksson A, von Schreeb J (2016). Effects of the West Africa Ebola virus disease on health-care utilization - a systematic review. Front Public Health.

[9] International Federation of the red Cross and red Crescent. Prevention and response to Sexual and Gender-Based Violence in COVID-19 – A Protection, Gender & Inclusion (PGI) Technical guidance note. https://reliefweb.int/sites/reliefweb.int/files/resources/IFRC-SGBV-COVID-19-Technical-Guidance-Note-FINAL_14May.pdf. Accessed Oct 26, 2020. May 14, 2020.

